# Copy numbers of the *S*-adenosylmethionine synthetase (*METK*) gene in 14 dogs with active *Leishmania infantum* infection

**DOI:** 10.1016/j.crpvbd.2026.100359

**Published:** 2026-02-10

**Authors:** Kathrin Sigel, Andreas Moritz, Melanie Hamann, Carlo Palizzotto, Eric Zini, Annabel Dalmau López, Britta Beck, Alexandra Kehl, Elisabeth Müller, Torsten J. Naucke, Yaarit Nachum-Biala, Gad Baneth, Ingo Schäfer

**Affiliations:** a Laboklin GmbH & Co. KG, Steubenstrasse 4, Bad Kissingen, Bavaria, 97688, Germany; b Clinical Pathology and Clinical Laboratory Diagnostics, Department of Veterinary Medicine, Justus-Liebig-University Giessen, Frankfurter Strasse 114, Giessen, Hesse, 35392, Germany; c Small Animal Clinic – Internal Medicine, Department of Veterinary Medicine, Justus-Liebig-University Giessen, Frankfurter Strasse 114, Giessen, Hesse, 35392, Germany; d Institute of Pharmacology and Toxicology, Faculty of Veterinary Medicine, Justus Liebig University Giessen, Schubertstrasse 81, Giessen, Hesse, 35392, Germany; e AniCura Istituto Veterinario Novara, Strada Provinciale 9, Granozzo con Monticello, NO, 28060, Italy; f Department of Animal Medicine, Production and Health, University of Padova, viale dell’Università 16, Legnaro, PD, 35020, Italy; g Clinic for Small Animal Internal Medicine, Vetsuisse Faculty, University of Zurich, Winterthurerstrasse 260, Zurich, 8057, Switzerland; h AniCura Mediterrani Hospital Veterinari, Carrer dels Fusters 3, Reus, 43204, Spain; i Koret School of Veterinary Medicine, The Hebrew University, Herzl 229, Rehovot, 76100, Israel

**Keywords:** Canines, Genetics, Leishmaniasis, Leishmaniosis, Polymerase chain reaction, Relapse

## Abstract

Relapses of canine leishmaniasis during allopurinol treatment are common and complicate the course of disease. *S*-adenosylmethionine synthetase (*METK*) gene copy numbers (CN) < 3.0 have been demonstrated in allopurinol-resistant *Leishmania infantum* strains *in vitro*, but its clinical impact *in vivo* is still unclear. This study included 14 dogs divided into two cohorts (C): Cohort one (CI): nine dogs (64%) with signs of disease relapse under allopurinol treatment; Cohort two (CII): five dogs (36%) recently diagnosed with active leishmaniasis prior to treatment. *Leishmania infantum* infection was confirmed by positive PCR testing. *METK* gene CN was quantified by droplet digital PCR. Complete blood counts and biochemical profiles were performed where suitable samples were available. *METK* gene CN ranged from 0.7 to 3.4 [CN < 3.0 (*n* = 13), 93%; CN = 3.4 (*n* = 1), 7%; CI: CN = 1.2–3.4; CII: CN < 2.0 each]. Clinicopathological abnormalities consistent with active leishmaniasis were observed in all dogs. Allopurinol is used for long-term management of canine leishmaniasis, therefore identification of resistance to allopurinol is crucial, especially in cases of clinical relapses. Leishmaniasis poses a zoonotic risk to humans so that the spread of parasites due to resistance should be considered regarding the One Health aspect and the All Species approach. In dogs recently diagnosed with active leishmaniasis not receiving allopurinol yet, resistant *L. infantum* strains may most likely be transmitted by sand flies. The threshold of *METK* gene CN < 3.0 *in vivo* seems to be questionable in individual cases.

## Highlights

• *METK* gene CN < 3.4 recorded in 9 dogs with clinical suspicion of resistance.
• *METK* gene CN < 3.0 recorded in 8 of the 9 dogs with clinical suspicion of resistance.
• *METK* gene CN < 2.0 recorded in 5 dogs prior to receiving allopurinol.
• Considering allopurinol resistance is crucial for further therapeutic management.
• Identifying dogs with resistance is crucial to avoid the spread of resistant strains.

## Graphical abstract

**Figure:**
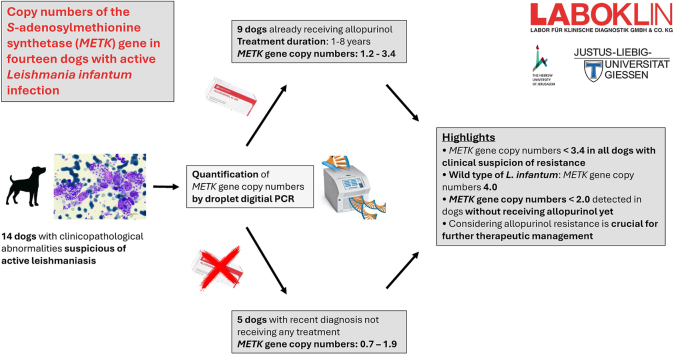

## 1 Introduction

Canine leishmaniasis is caused by the protozoan species *Leishmania infantum* and is transmitted by phlebotomine sand flies in the Mediterranean countries in Europe (Van Bockstal et al., 2020; Maia et al., 2023). In addition to vector transmission, blood donations (de Freitas et al., 2006), mating (Naucke and Lorentz, 2012), and intrauterine infections (Naucke and Lorentz, 2012) have been described as additional modes of transmission. Canines are considered the primary reservoir for human infection with *L. infantum* and therefore pose a zoonotic risk, particularly to children and immunocompromised people (Dantas-Torres, 2007; Quinnell and Courtenay, 2009; Richter et al., 2011). The import of dogs across borders, increasing international travel, and climate change contribute to the spread of disease to non-endemic countries, like Germany.

Clinical signs of canine leishmaniasis are largely unspecific, ranging from skin lesions (81–89%) (Koutinas et al., 1999; Perego et al., 2014) to generalized lymph adenomegaly (56–90%) (Slappendel, 1988; Ciaramella et al., 1997), splenomegaly (10–53%) (Slappendel, 1988; Ciaramella et al., 1997; Koutinas et al., 1999), ocular lesions (11–81%) (Slappendel, 1988; Ciaramella et al., 1997; Koutinas et al., 1999; Pena et al., 2000), wasting (25–64%) (Slappendel, 1988; Ciaramella et al., 1997; Koutinas et al., 1999), pale mucous membranes (58%) (Slappendel, 1988; Ciaramella et al., 1997), and lethargy (10–60%) (Slappendel, 1988; Koutinas et al., 1999; Solano-Gallego et al., 2011). The manifestation and severity of clinical signs is contingent on age, breed, co-infections, and the immunological condition of dogs at the time of infection (Solano-Gallego et al., 2011). A pronounced cell-mediated immune response with an increase in CD8+ lymphocytes is associated with subclinical infection (Garcia-Castro et al., 2022; Ivanescu et al., 2023), whereas dogs with a dominant humoral response are more likely to develop severe clinical signs and high blood parasitemia (Solano-Gallego et al., 2016). Mild normochromic, normocytic anemia (7–73%) (Slappendel, 1988; Ciaramella et al., 1997; Koutinas et al., 1999; Solano-Gallego et al., 2011; Baxarias et al., 2023) and thrombocytopenia (3–50%) (Slappendel, 1988; Ciaramella et al., 1997; Koutinas et al., 1999; Solano-Gallego et al., 2011; Baxarias et al., 2023), hyperproteinemia (63–73%) (Slappendel, 1988; Ciaramella et al., 1997; Koutinas et al., 1999; Solano-Gallego et al., 2011), hyperglobulinemia (76–100%) (Slappendel, 1988; Ciaramella et al., 1997; Koutinas et al., 1999; Solano-Gallego et al., 2011), proteinuria (36–85%) (Slappendel, 1988; Ciaramella et al., 1997; Koutinas et al., 1999; Solano-Gallego et al., 2011; Baxarias et al., 2023), and renal azotemia (2–45%) (Slappendel, 1988; Ciaramella et al., 1997; Koutinas et al., 1999; Solano-Gallego et al., 2011; Baxarias et al., 2023) are the most frequently seen laboratory abnormalities.

*Leishmania infantum* infections are diagnosed by quantitative serological methods like enzyme-linked immunosorbent assay (ELISA, 94–100% sensitivity, 52–100% specificity, depending on the antigen), or immunofluorescence antibody test (IFAT, up to 90% sensitivity in clinical cases, 64–100% specificity) (Mettler et al., 2005; Ferreira Ede et al., 2007). The presence of high antibody levels alongside corresponding clinical signs and laboratory abnormalities is indicative of disease (Solano-Gallego et al., 2011).

Confirmatory polymerase chain reaction (PCR) for parasitic deoxyribonucleic acid (DNA) offers high sensitivity, especially when using kinetoplast DNA minicircles or small subunit rRNA genes as targets (Maia and Campino, 2008; Paltrinieri et al., 2010). EDTA blood samples from infected dogs often contain lower yields of *Leishmania* and are therefore less sensitive than the gold standard of bone marrow (Paltrinieri et al., 2010).

Therapeutic options can be classified as leishmanicidal (mostly meglumine antimoniate or miltefosine) or leishmaniostatic (allopurinol). Furthermore, the utilization of herbal (e.g. artemisinin, alpha-bisabolol) or immunostimulatory medications (e.g. domperidone) as a supplement has been documented (Schäfer et al., 2022). The standard long-term management of canine leishmaniasis is based on allopurinol (Solano-Gallego et al., 2011). In contrast, this medication is rarely used for the treatment of leishmaniasis in humans, where it is more commonly seen in gout prevention (Pfaller and Marr, 1974; Schäfer et al., 2022).

Clinical relapse of canine leishmaniasis, characterized by the recurrence of clinical signs and/or clinicopathological abnormalities after apparent disease recovery, is common and complicates the course of the disease (Solano-Gallego et al., 2011; Sarquis et al., 2024). It has been suggested that members of the genus *Leishmania* may adapt to environmental triggers through their inherent genomic instability (Marti-Carreras et al., 2022; Black et al., 2023). Resulting genomic rearrangements may include mosaic aneuploidies or gene copy number variations, regulating gene transcription (Ubeda et al., 2014; Laffitte et al., 2016; Marti-Carreras et al., 2022; Wagner et al., 2025). Yasur-Landau et al. (2016, 2018) associated clinical suspicion of treatment failure with allopurinol resistance in dogs infected with *L. infantum* and reported a reduction in copy number (CN) of the parasitic *S*-adenosylmethionine synthetase (*METK*) gene < 3.0. Copy number variations of the *METK* gene of *L. infantum* were also identified and further investigated by nanopore sequencing (Marti-Carreras et al., 2022). It was assumed, amongst other theories, that the incorporation of allopurinol into energetic nucleotides inhibits the *S*-adenosylmethionine synthetase, a crucial enzyme in the metabolism of *Leishmania* (Yasur-Landau et al., 2018). Consequently, reduced *METK* gene CN led to lower amounts of the *S*-adenosylmethionine synthetase, which might result in reduced inhibitory effects of allopurinol on parasite growth (Yasur-Landau et al., 2018).

*METK* gene CN of < 3.0 in dogs with clinical suspicion of allopurinol resistance is most likely an underlying cause of treatment failure. As transmission potential of antimony-resistant *L. infantum* strains by sand flies was reported (Seblova et al., 2014; Van Bockstal et al., 2020), it is suggested that resistance to allopurinol additionally occurs in dogs with recent *L. infantum* diagnosis not undergoing treatment in the past. The aim of this study was to detect allopurinol resistance in dogs infected with *L. infantum* based on the quantification of *METK* gene CN.

## 2 Materials and methods

### 2.1 Study population

Dogs were identified by screening results of a monitoring profile for dogs with leishmaniasis conducted by the Laboklin laboratory (Bad Kissingen, Germany) on a fee-for-service basis, and on laboratory abnormalities suspicious for clinical relapse of disease between September 2023 and October 2025. Further analysis was performed on a research basis on surplus material left over after diagnostics, and the veterinarians were contacted to supply clinical records for the dogs included. Routine check-ups on dogs with documented or recently diagnosed infections with *L. infantum* were additionally included.

A total of 14 dogs fulfilled the criteria. One of these dogs was already described in a case report (Schäfer et al., 2024). Signalment is summarized in Table 1.

**Table 1:** Signalment, origin, treatment, and *S*-adenosyl-methionine synthetase (*METK*) gene copy numbers (CN) in 14 dogs with active *Leishmania infantum* infection.

ID	Breed	Sex	Age in years	Residence	Import history^a^	Allopurinol administration^b^	Previously added therapy^c^	Corticosteroid treatment	*METK* gene CN
**Cohort I: Dogs with clinical suspicion of active leishmaniasis under allopurinol treatment (*****n***** = 9, 64%)**									
1	Akita Inu	F	7	France (Bordeaux)	–	4	Meglumine-antimoniate (2 cycles)	No	<3.0
2	Mix	F	10	Israel	–	8	Miltefosine (1 cycle); immune stimulants	No	2.1
3	Podenco	F	3	Germany	Spain (3 years)	1	Miltefosine (2 cycles); immune stimulants	No	2.6
4	Mix	M	4	Germany	Greece (3 years)	2	–	No	1.2
5	Golden Retriever	M	4	France (Toulouse)	–	1	Meglumine-antimoniate (2 cycles); miltefosine (1 cycle)	No	3.4
6	Jack Russell Terrier	M	6	Germany	Greece (4 years)	4	Miltefosine (2 cycles); immune stimulants	No	2.1
7	Doberman	F	8	Germany	Greece (6 years)	2	Immune stimulants	No	2.0
8	Labrador Retriever	F	9	Italy	–	2	Meglumine-antimoniate (2 cycles); miltefosine (2 cycles); immune stimulants	Yes	2.2
9	Mix	M	5	Germany	na^d^	3	Immune stimulants	No	1.2
**Cohort II: Dogs recently diagnosed with active leishmaniasis not receiving allopurinol yet (*****n***** = 5, 36%)**									
10	Mix	M	6	Germany	Bulgaria (3 years)	0	–	Yes	1.1
11	Jack Russell-Terrier-Mix	M	6	Germany	Greece (3 years)	0	–	No	1.9
12	Ratonero-Mix	F	4	Germany	Spain (3 years)	0	–	No	1.8
13	English Setter	F	7	Germany	Spain (1 year)	0	–	No	0.7
14	Boxer	M	1	Italy	–	0	–	Yes	1.4

*Abbreviations*: CN, copy number; F, female; M, male; *METK*, *S*-adenosyl methionine synthase.

a Import history (timeframe from import to Germany and testing for allopurinol resistance in years).

b Timeframe of oral administration of allopurinol between start of therapy and testing for allopurinol resistance (in years) - equivalent to the first time of diagnosis in each case (timeframe between first diagnosis of leishmaniasis and testing for allopurinol resistance (in years)).

c Additional therapy between the first diagnosis of leishmaniasis and testing for allopurinol resistance, no additional leishmanicidal therapy at the time of testing for resistance in all cases.

d Import history unknown.

### 2.2 Hematological and biochemical analysis

Samples were submitted to the laboratory Laboklin (Bad Kissingen, Germany) by overnight shipping and included EDTA blood (12/14 dogs), as well as serum/plasma samples (10/14 dogs) and urine samples (8/14 dogs). Complete blood counts (Sysmex XN-V analyzer, Sysmex Deutschland, Norderstedt, Germany), biochemical profiles (Cobas 8000, Roche Diagnostics GmbH, Mannheim, Germany), and serum protein capillary electrophoresis (Sebia Minicap and Sebia Capillarys 3 Octa, Sebia, Mainz, Germany) were performed on blood samples. Urine was analyzed on the Cobas u601 (Roche Diagnostics GmbH, Mannheim, Germany), microscopically examined, and a urine protein-to-creatinine ratio (UPC) was calculated on the Cobas 8000 (Roche Diagnostics GmbH, Mannheim, Germany). Hematological and biochemical parameters are summarized in Table 2.

**Table 2:** Clinical signs and laboratory test abnormalities in 14 dogs with active *Leishmania infantum* infection.

ID	Clinical signs						Laboratory test abnormalities							
	Dermatological signs	Lymph adenomegaly	Epistaxis	Cystitis	Weight loss	Fever	Anemia^a^	Thrombocytopenia^b^	Leukocytes^c^	Hyperproteinemia^d^	Hypergammaglobulinemia^e^	Azotemia^f^	CRP^g^	UPC^h^
**Cohort I: Dogs with clinical suspicion of active leishmaniasis under allopurinol treatment (*****n***** = 9, 64%)**														
1	No	Yes	No	No	No	No	+	+	0	+	+	+	+	++
2	Yes	Yes	No	Yes	No	No	+	–	na	+++	na	+	na	na
3	Yes	No	No	No	No	No	–	–	0	–	–	–	+	–
4	Yes	Yes	Yes	No	No	No	+++	–	+	+++	+++	–	++	+
5	Yes	Yes	Yes	No	No	No	++	–	–	+++	na	–	na	na
6	Yes	No	No	Yes	No	Yes	+	–	+	+++	++	++	+++	++
7	Yes	Yes	No	No	Yes	No	+	++	–	+	+	–	++	++
8	No	Yes	No	Yes	No	No	++	–	0	+++	na	++	na	++
9	Yes	No	No	No	No	No	++	+	–	++	+	+	na	na
**Cohort II: Dogs recently diagnosed with active leishmaniasis not receiving allopurinol yet (*****n***** = 5, 36%)**														
10	No	No	Yes	Yes	No	Yes	+	–	0	–	+	+	–	+++
11	No	Yes	No	No	No	No	+	–	0	+	na	–	++	na
12	Yes	No	No	No	No	No	–	–	0	–	–	–	–	–
13	No	Yes	No	No	No	No	–	+	0	–	–	–	na	na
14	No	Yes	No	Yes	No	No	+	–	+	–	+	++	+	++

*Abbreviations*: CRP, c-reactive protein; UPC, urine protein-to-creatinine ratio; na, no information available.

a Anemia: –, absent (hematocrit: > 0.43 l/l); +, mild (0.30–0.43 l/l); ++, moderate (0.20–0.29 l/l); +++, marked (< 0.20 l/l).

b Thrombocytopenia: –, absent (> 149 G/l); +, mild (90–149 G/l); ++, moderate (30–90 G/l); +++, marked (< 30 G/l).

c Leukocytes: 0, in reference range (6.0–12.0 G/l); –, leukopenia (< 6.0 G/l); +, leukocytosis (> 12.0 G/l).

d Hyperproteinemia: –, absent (< 75 g/l total protein); +, mild (75–80 g/l); ++, moderate (80–85 g/l); +++, marked (> 85 g/l).

e Hypergammaglobulinemia: –, absent (< 15 g/l gamma globulins); +, mild (15–25 g/l); ++, moderate (25–35 g/l); +++, marked (> 35 g/l).

f Azotemia: –, absent (< 125 μmol/l creatinine); +, mild (125–250 μmol/l); ++, moderate (251–440 μmol/l); +++, marked (> 440 μmol/l).

g CRP: –, not elevated (< 15 mg/l); +, mildly elevated (15–50 mg/l); ++, moderately elevated (51–100 mg/l); +++, highly elevated (> 100 mg/l).

h UPC: –, not elevated (< 0.5); +, mildly elevated (0.5–0.9); ++, moderately elevated (1.0–5.0); +++, highly elevated (> 5.0).

### 2.3 Diagnosis of infection with *L. infantum*

DNA was extracted from blood (*n* = 7), lymph node aspirates (*n* = 4), bone marrow (*n* = 2), and skin crust samples (*n* = 1), using the MagNA Pure 96 DNA and Viral NA Small Volume Kit (Roche Diagnostics GmbH, Mannheim, Germany) following the manufacturer's instructions. TaqMan real-time PCR for *L. infantum* (target: kinetoplast minicircle DNA, primers: 5′-AAC TTT TCT GGT CCT CCG GGT AG-3′ and 5′-ACC CCC AGT TTC CCG CC-3′; probe: 5′-FAM-AAA AAT GGG TGC AGA AAT NFQMGB-3′) (Francino et al., 2006) was performed using the DNA/RNA Process Control Detection Kit (Roche Diagnostics GmbH, Mannheim, Germany) on a thermocycler (LightCycler 96, Roche Diagnostics GmbH, Mannheim, Germany). A cycle threshold of below 35 was set. Negative, positive, and extraction controls were included in each run (DNA/RNA Process Control Detection Kit, Roche Diagnostics GmbH, Mannheim, Germany).

Enzyme-linked immunosorbent assay (ELISA) testing (Gold Standard Diagnostics VetLine *Leishmania* ELISA, Gold Standard Diagnostics Frankfurt GmbH, Dietzenbach, Germany, > 11 technical units (LE) positive) was performed for serological detection of *Leishmania* spp. infection.

### 2.4 Quantification of *METK* gene copy numbers

Blood samples with high cycle thresholds (CT > 27.0) in real-time PCR upon arrival were prepared for cultivation by adding 10 ml of cultivation medium. After incubation, DNA was extracted from the cultured samples as described above. A second PCR was performed following culturing to compare cycle thresholds before and after cultivation. Samples with low cycle thresholds (Ct < 25.0) (*n* = 4) after cultivation were prepared for *METK* CN quantification, whereas samples with low residual parasitic load were excluded from the study, as there was insufficient DNA for *METK* CN quantification. Droplet digital PCR was performed for the quantification of the *METK* gene by the usage of Supermix, Droplet Generator, C1000 Touch Thermal Cycler and Droplet Reader (BioRad Laboratories, Inc., Feldkirchen, Germany) at the laboratory Laboklin (Bad Kissingen, Germany). Glyceraldehyde-3-phosphate dehydrogenase (*GAPDH*) was used as a reference gene. Samples from dogs negatively tested for *Leishmania* were used as negative controls to exclude cross-reactivity with canine DNA. Accuracy was validated by measuring the *METK* gene CN in the WHO reference strain MHOM/TN/80/IPT-1, characterized by having a single *METK* copy (Marti-Carreras et al., 2022). The validity of the test system for *METK* gene quantification was confirmed through the execution of inter- and intra-assay tests, demonstrating the test system as robust, with a variation coefficient of less than 0.15.

## 3 Results

A background travel history was available for 13 of the 14 dogs (93%) and in all cases included a region with endemic *L. infantum* (Baneth et al., 2022; Maia et al., 2023). The sex distribution was uniform, with 7 female (50%) and 7 male (50%) dogs. Eight of the 14 dogs were classified as purebred (57%), and 6 were identified as mixed breeds (43%). Infection with *L. infantum* was confirmed by PCR testing in all 14 dogs. The dogs were divided into two cohorts according to whether they had previously been treated with allopurinol at the time of resistance testing (Table 1).

Dogs that had previously received allopurinol (9/14 dogs; 64%) were included in Cohort I (CI). Individual treatment durations (median 2.0 years, standard deviation 2.1 years, minimum 1.0 year, maximum 8.0 years) are shown in Table 1. *METK* gene CN in this cohort ranged from 1.2 to 3.4 (Table 1). In one dog (ID: 5, 7%), the *METK* gene CN exceeded the suggested *in vitro* threshold of 3.0, associated with allopurinol resistance (Yasur-Landau et al., 2018). Each of the 9 dogs in CI was presented to their attending veterinarians with clinicopathological abnormalities while undergoing allopurinol maintenance therapy, all consistent with active canine leishmaniasis. Dermatological abnormalities (7/9 dogs, 78%) were the most common clinical sign in CI, followed by lymphadenomegaly (6/9 dogs, 67%). More rarely observed were hematuria or bacteriuria indicative of cystitis (3/9 dogs, 33%), epistaxis (2/9 dogs, 22%), fever (defined as rectal temperature > 39.0 °C, 1/9 dogs, 11%) and weight loss (1/9 dogs, 11%) (Table 2). The most common hematological anomalies were anemia and hyperproteinemia (8/9 dogs, 89% for both). Anemia was mild in half of the cases (4/8 dogs, 50%), whereas hyperproteinemia was moderate to severe in most of the cases (6/8 dogs, 75%). Hypergammaglobulinemia occurred in five of six dogs (5/6, 83%), in which serum protein electrophoresis was performed. Other hematological anomalies included thrombocytopenia (3/9, 33%) and elevated C-reactive protein (5/5 dogs analyzed, 100%) and UPC (5/6 dogs analyzed, 83%). Azotemia defined by an elevation of the serum creatinine level according to the guidelines of the International Renal Interest Society (IRIS) was detected in about half of the dogs (5/9 dogs, 56%) (Table 2).

Dogs that had not been treated for leishmaniasis prior to being tested for resistance were included in Cohort II (5/14 dogs, 36%). All five dogs were recently clinically diagnosed with active leishmaniasis by their treating veterinarians. The quantification of the *METK* gene CN in CII exclusively showed CN < 2.0, with an individual range from 0.7 to 1.9 (Table 1). Clinicopathological abnormalities indicative of active leishmaniasis were observed in all five dogs in CII, albeit overall less frequently than in CI. In this group, the most prevalent clinical sign was lymphadenomegaly (3/5 dogs, 60%), followed by signs indicative of cystitis as mentioned above (2/5 dogs, 40%), dermatological signs, epistaxis and fever (1/5 dogs respectively, 20%). Like in CI, anemia was identified as the most frequent hematological abnormality (3/5 dogs, 60%), being mild in all cases (100%). C-reactive protein was analyzed in four dogs (4/5, 80%) and was elevated in half of them (2/4, 50%), while UPC was analyzed in three dogs (3/5, 60%) and was elevated in two of them (2/3, 67%). Hyperproteinemia and hypergammaglobulinemia, occurred in one of five dogs (20%) and two of four dogs (50%), in which serum protein electrophoresis was performed.

At the time of resistance testing, three of 14 dogs (21%, one in CI and two in CII) received corticosteroids (Table 1).

## 4 Discussion

This is currently the only study analyzing *METK* gene copy number variations *in vivo* in a larger number of dogs with active leishmaniasis. Droplet digital PCR was established as a fast and reliable diagnostic method for routinely determining the *METK* gene CN of *L. infantum*, provided there was sufficient parasite DNA available in the sample material.

Disease relapses necessitate prompt intervention to prevent exacerbation of leishmaniasis and accelerated proliferation of parasites (Solano-Gallego et al., 2011; Sarquis et al., 2024). To adapt treatments accordingly, investigations of the underlying cause for treatment failure must be quick, accurate, and standardizable. The findings of Cohort I (CI) in this study suggest that in dogs treated with allopurinol and *METK* gene CN < 3.0, therapeutic options must be reevaluated, and cases with CN of 3.0–4.0 should be monitored for clinicopathological signs of relapse.

Furthermore, it is of epidemiological interest to identify carriers of resistant *Leishmania* strains and to avoid further spread of resistant strains by sand flies, not just to other dogs but also to other animals and humans. It is important to note that there are currently no drugs available that can lead to complete parasite clearance in dogs (Travi et al., 2018; Wagner et al., 2025). Allopurinol remains the preferred drug for long-term infection control. As allopurinol is not routinely used in the treatment of humans with leishmaniasis, resistant strains do not pose a direct risk to humans. However, dogs infected with allopurinol-resistant strains may show higher parasite loads and serve as an important infection reservoir to humans (Sarquis et al., 2024; Wagner et al., 2025). This poses a risk on a larger scale, especially regarding the One Health aspect and All Species approach (Wagner et al., 2025).

*METK* gene CNs < 3.0 were detected in 13 dogs, and CN = 3.4 in one dog with active leishmaniasis. The dogs were disseminated in two cohorts, including Cohort I (CI) with nine dogs (64%) showing clinical suspicion of disease relapse while undergoing long-term management with allopurinol. Therefore, allopurinol resistance is strongly suspected as the underlying cause of treatment failure and recurring active leishmaniasis. In Cohort II (CII), including five of 14 dogs (36%) with a recent diagnosis of active leishmaniasis not receiving allopurinol yet, the infection by resistant *Leishmania* strains seems most likely.

If available, travel history for all dogs suggested current or past exposure to sand flies in an endemic region (Baneth et al., 2022; Maia et al., 2023). The data therefore do not support any suggestions of the occurrence of autochthonous infections in a currently non-endemic country like Germany. No anomalies were observed in terms of sex frequency or breed distribution. While several dogs (21%) in this study cohort received prednisolone at the time of resistance testing, there are insufficient data to investigate a possible alteration of *METK* gene CN due to the administration of steroids, and further research may be warranted.

Cutaneous lesions and lymphadenomegaly were observed as the most frequent clinical signs in this study, and anemia and hyperproteinemia were much more commonly observed than thrombocytopenia, which is consistent with the literature (Slappendel, 1988; Koutinas et al., 1999; Solano-Gallego et al., 2011).

All nine dogs included in Cohort I showed clinicopathological signs indicative of an active infection with *L. infantum* at the time of resistance testing, despite ongoing allopurinol therapy. Therefore, clinical suspicion of allopurinol resistance was assumed and defined as the occurrence of at least one clinical sign and at least one hematological and biochemical abnormality associated with active leishmaniasis, despite maintenance therapy with allopurinol. In addition to the manifestation of clinicopathological abnormalities indicative of disease relapse, *METK* gene CNs < 3.0 were observed in eight of the nine dogs (89%). These results are consistent with previously published *in vitro* data by Yasur-Landau et al. (2018), demonstrating a deletion of one copy of the *METK* gene in allopurinol-resistant *L. infantum* strains in comparison to the *L. infantum* wild type characterized with a *METK* gene CN of 4.0.

However, *METK* gene quantification in one dog (ID: 5) with a clinical suspicion of allopurinol resistance revealed a CN above the previously suggested threshold of < 3.0 associated with allopurinol resistance *in vitro* (Yasur-Landau et al., 2018). At 3.4, the *METK* gene CN was > 3.0 but still below the wild-type *METK* gene CN of 4.0 (Yasur-Landau et al., 2018). This makes an *in vivo* threshold of the *METK* gene CN < 3.0 as a suggested diagnostic cut-off in individual cases worth discussing. It is important to note that this dog was residing in an endemic area, rendering an infection with a novel strain of *L. infantum* shortly prior to resistance testing possible. Hence, the result of the quantification of the *METK* gene CN in the mentioned case could be discussed as a composite of the CN of resistant and susceptible *L. infantum* strains. Furthermore, the sample material in this case was a lymph node biopsy, and allopurinol concentration and distribution in different tissues *in vivo* have not been well documented. This means that there could possibly be several strains coexisting in macrophages within lymph nodes. Lastly, the duration of the allopurinol treatment prior to resistance testing was notably shorter in this case than the median treatment duration of the study population. Further research is needed to investigate the development of allopurinol resistance, which could be initiated in different parasites at varying rates.

The second cohort (CII) in this study consisted of five dogs recently diagnosed with *L. infantum* infection, which were not treated with allopurinol at the time of resistance testing. All five dogs showed at least one clinicopathological abnormality consistent with active leishmaniasis. Interestingly, in all five dogs *METK* gene CN wereas < 2.0. No plausible explanation for the lower *METK* gene CN in CII in comparison to CI could be demonstrated yet. While import from a Mediterranean country in most of the cases means treatment with allopurinol prior to arrival in Germany cannot be excluded, transmission of antimony-resistant strains of *L. infantum* in sand fly vectors has also been described in the literature (Seblova et al., 2014; Van Bockstal et al., 2020) and therefore remains a possibility in these cases. It is of significant scientific and clinical interest to further monitor these cases to assess the efficacy of allopurinol in the subsequent therapeutic management. Overall, the transmission of allopurinol-resistant parasite strains from dogs undergoing allopurinol therapy *via* sand flies is highly probable and should be considered at the first time of diagnosis of leishmaniasis. In view of the findings, testing for allopurinol resistance at the initial point of diagnosis may support the evaluation of the efficacy of different treatment options. This, in turn, could improve the management of canine leishmaniasis by preventing therapy failure, which might occur in case of disease relapse and worsens the prognosis of the individual patient (Sarquis et al., 2024).

In human medicine, genotypic changes in *L. infantum* parasites associated with drug resistance have been shown to be unstable during *in vivo* passaging in both vertebrate and non-vertebrate hosts (Van den Kerkhof et al., 2020). Drug-induced, non-fixed genetic alterations might therefore be reversible in the absence of drug pressure (Van den Kerkhof et al., 2020). Consequently, long-term follow-ups of *METK* gene CN in cases of suspected allopurinol resistance are essential to prove an upregulation of the *METK* gene CN in the absence of allopurinol administration.

The limitations of this study included that co-infections with other vector-borne pathogens and comorbidities potentially may have influenced the clinicopathological presentation in the dogs described in this study. However, the impact of co-infections or underlying diseases on the quantification of the *METK* gene CN, performed by validated molecular testing as described above, is assumed to be negligible. As droplet digital PCR analysis requires high quantities of parasite DNA to provide reliable results at the quantification of the *METK* gene CN, testing is limited to sample material with high parasite loads. For this method, bone marrow would be considered the gold standard material, with spleen, lymph node aspirates, or skin crusts as reasonable alternatives. EDTA blood samples with low parasite load after cultivation needed to be excluded from the study, as droplet digital PCR could not provide reliable results. Quantification of *METK* gene CN of dogs with efficient allopurinol therapy was therefore not possible yet, due to low parasite loads. The incorporation of a corresponding control cohort remains unfeasible at this time and should be the focus of future research. Cases with unavailable clinical information were also excluded. Overall, the limitation of the detection method resulted in stringent inclusion criteria and, consequently, relatively low numbers of cases in both cohorts. This again limits the statistical analysis of the quantification of *METK* gene CN as well as of the clinicopathological abnormalities. Therefore, only descriptive statistics were provided in the study.

## 5 Conclusions

In dogs with a clinical suspicion of resistance to ongoing allopurinol treatment for *L. infantum* infections, *METK* gene CNs < 3.0 were detected in eight of nine dogs (89%) and a CN = 3.4 (11%) in the remaining dog. Therefore, a threshold of the *METK* gene CN < 3.0 *in vivo* as a possible marker of resistance seems questionable in individual cases. If resistance is clinically suspected, *METK* gene CN below the wild type of *L. infantum* (< 4.0) should additionally be considered in further management of treatment in dogs with canine leishmaniasis. Transmission of resistant *L. infantum* strains at the initial point of infection appears to be probable and needs to be taken into consideration when leishmaniasis is recently diagnosed. Routine testing for resistance by quantifying the *METK* gene CN can therefore be considered beneficial at the initial point of diagnosis or whenever there is a suspicion of disease relapse. In individual cases, treatments can be adapted accordingly to prevent further disease relapse and improve prognosis. Detection of allopurinol-resistant *Leishmania* strains is of high epidemiological interest to avoid the spread of resistant strains transmitted by sand flies in endemic countries.

## Ethical approval

Not applicable.

## CRediT authorship contribution statement

**Kathrin Sigel:** Investigation, Visualization, Writing - original draft. **Andreas Moritz:** Supervision, Writing - review & editing. **Melanie Hamann:** Writing - review & editing. **Carlo Palizzotto:** Writing - review & editing. **Eric Zini:** Writing - review & editing. **Annabel Dalmau López:** Writing - review & editing. **Britta Beck:** Investigation, Writing - review & editing. **Alexandra Kehl:** Investigation, Writing - review & editing. **Elisabeth Müller:** Writing - review & editing. **Torsten J. Naucke:** Writing - review & editing. **Yaarit Nachum-Biala:** Writing - review & editing. **Gad Baneth: Writing - review & editing. Ingo Schäfer:** Conceptualization, Supervision, Writing - review & editing.

## Funding

This research did not receive any specific grant from funding agencies in the public, commercial, or not-for-profit sectors.

## Declaration of competing interests

Kathrin Sigel, Britta Beck, Alexandra Kehl, Torsten J. Naucke, and Ingo Schäfer are employees, and Elisabeth Müller is the CEO of Laboklin GmbH & Co. KG, offering the mentioned tests on a fee-for-service basis to veterinarians. Andreas Moritz, Melanie Hamann, Gad Baneth, and Ingo Schäfer declare that they repeatedly lectured for and/or acted as consultants for diagnostic and (veterinary) pharmaceutical companies. Andreas Moritz and Melanie Hamann have previous and ongoing research collaborations with various diagnostic and (veterinary) pharmaceutical companies. The other authors declare that they have no known competing financial interests or personal relationships that could have appeared to influence the work reported in this paper.

## Data Availability

The data supporting the conclusions of this article are included in this published article.

## References

[bib1] Baneth G., Nachum-Biala Y., Adamsky O., Gunther I. (2022). *Leishmania tropica* and *Leishmania infantum* infection in dogs and cats in central Israel. Parasites Vectors.

[bib2] Baxarias M., Jornet-Rius O., Donato G., Mateu C., Alcover M.M., Pennisi M.G., Solano-Gallego L. (2023). Signalment, immunological and parasitological status and clinicopathological findings of *Leishmania*-seropositive apparently healthy dogs. Animals.

[bib3] Black J.A., Reis-Cunha J.L., Cruz A.K., Tosi L.R.O. (2023). Life in plastic, it’s fantastic! How *Leishmania* exploit genome instability to shape gene expression. Front. Cell. Infect. Microbiol..

[bib4] Ciaramella P., Oliva G., Luna R.D., Gradoni L., Ambrosio R., Cortese L. (1997). A retrospective clinical study of canine leishmaniasis in 150 dogs naturally infected by *Leishmania infantum*. Vet. Rec..

[bib5] Dantas-Torres F. (2007). The role of dogs as reservoirs of *Leishmania* parasites, with emphasis on *Leishmania* (*Leishmania*) *infantum* and *Leishmania* (*Viannia*) *braziliensis*. Vet. Parasitol..

[bib6] de Freitas E., Melo M.N., da Costa-Val A.P., Michalick M.S. (2006). Transmission of *Leishmania infantum via* blood transfusion in dogs: potential for infection and importance of clinical factors. Vet. Parasitol..

[bib7] Ferreira Ede C., de Lana M., Carneiro M., Reis A.B., Paes D.V., da Silva E.S. (2007). Comparison of serological assays for the diagnosis of canine visceral leishmaniasis in animals presenting different clinical manifestations. Vet. Parasitol..

[bib8] Francino O., Altet L., Sanchez-Robert E., Rodriguez A., Solano-Gallego L., Alberola J. (2006). Advantages of real-time PCR assay for diagnosis and monitoring of canine leishmaniosis. Vet. Parasitol..

[bib9] Garcia-Castro A., Egui A., Thomas M.C., Lopez M.C. (2022). Humoral and cellular immune response in asymptomatic dogs with visceral leishmaniasis: a review. Vaccines.

[bib10] Ivanescu L., Andronic B.L., Grigore-Hristodorescu S., Martinescu G.V., Mindru R., Miron L. (2023). The immune response in canine and human leishmaniasis and how this influences the diagnosis - a review and assessment of recent research. Front. Cell. Infect. Microbiol..

[bib11] Koutinas A.F., Polizopoulou Z.S., Saridomichelakis M.N., Argyriadis D., Fytianou A., Plevraki K.G. (1999). Clinical considerations on canine visceral leishmaniasis in Greece: a retrospective study of 158 cases (1989–1996). J. Am. Anim. Hosp. Assoc..

[bib12] Laffitte M.N., Leprohon P., Papadopoulou B., Ouellette M. (2016). Plasticity of the *Leishmania* genome leading to gene copy number variations and drug resistance. F1000Res.

[bib14] Maia C., Conceicao C., Pereira A., Rocha R., Ortuno M., Munoz C. (2023). The estimated distribution of autochthonous leishmaniasis by *Leishmania infantum* in Europe in 2005–2020. PLoS Negl. Trop. Dis..

[bib13] Maia C., Campino L. (2008). Methods for diagnosis of canine leishmaniasis and immune response to infection. Vet. Parasitol..

[bib15] Marti-Carreras J., Carrasco M., Gomez-Ponce M., Noguera-Julian M., Fisa R., Riera C. (2022). Identification of *Leishmania infantum* epidemiology, drug resistance and pathogenicity biomarkers with nanopore sequencing. Microorganisms.

[bib16] Mettler M., Grimm F., Capelli G., Camp H., Deplazes P. (2005). Evaluation of enzyme-linked immunosorbent assays, an immunofluorescent-antibody test, and two rapid tests (immunochromatographic-dipstick and gel tests) for serological diagnosis of symptomatic and asymptomatic *Leishmania* infections in dogs. J. Clin. Microbiol..

[bib17] Naucke T.J., Lorentz S. (2012). First report of venereal and vertical transmission of canine leishmaniosis from naturally infected dogs in Germany. Parasites Vectors.

[bib18] Paltrinieri S., Solano-Gallego L., Fondati A., Lubas G., Gradoni L., Castagnaro M. (2010). Guidelines for diagnosis and clinical classification of leishmaniasis in dogs. J. Am. Vet. Med. Assoc..

[bib19] Pena M.T., Roura X., Davidson M.G. (2000). Ocular and periocular manifestations of leishmaniasis in dogs: 105 cases (1993–1998). Vet. Ophthalmol..

[bib20] Perego R., Proverbio D., Bagnagatti De Giorgi G., Spada E. (2014). Prevalence of dermatological presentations of canine leishmaniasis in a nonendemic area: a retrospective study of 100 dogs. Vet. Med. Int..

[bib21] Pfaller M.A., Marr J.J. (1974). Antileishmanial effect of allopurinol. Antimicrob. Agents Chemother..

[bib22] Quinnell R.J., Courtenay O. (2009). Transmission, reservoir hosts and control of zoonotic visceral leishmaniasis. Parasitology.

[bib23] Richter J., Hanus I., Haussinger D., Loscher T., Harms G. (2011). Mucosal *Leishmania infantum* infection. Parasitol. Res..

[bib24] Sarquis J., Raposo L.M., Sanz C.R., Montoya A., Barrera J.P., Checa R. (2024). Relapses in canine leishmaniosis: risk factors identified through mixed-effects logistic regression. Parasites Vectors.

[bib25] Schäfer I., Faucher M., Nachum-Biala Y., Ferrer L., Carrasco M., Kehl A. (2024). Evidence for *in vivo* resistance against allopurinol in a dog infected with *Leishmania infantum* by reduction in copy numbers of the *S*-adenosylmethionine synthetase (*METK*) gene. Parasites Vectors.

[bib26] Schäfer I., Müller E., Naucke T.J. (2022). Canine leishmaniosis: an update regarding diagnostics, therapeutic approaches, and monitoring. Tierarztl. Prax. Ausg. K Kleintiere Heimtiere.

[bib27] Seblova V., Oury B., Eddaikra N., Ait-Oudhia K., Pratlong F., Gazanion E. (2014). Transmission potential of antimony-resistant *Leishmania* field isolates. Antimicrob. Agents Chemother..

[bib28] Slappendel R.J. (1988). Canine leishmaniasis. A review based on 95 cases in the Netherlands. Vet. Q..

[bib29] Solano-Gallego L., Miro G., Koutinas A., Cardoso L., Pennisi M.G., Ferrer L. (2011). LeishVet guidelines for the practical management of canine leishmaniosis. Parasites Vectors.

[bib30] Solano-Gallego L., Montserrrat-Sangra S., Ordeix L., Martinez-Orellana P. (2016). *Leishmania infantum*-specific production of IFN-gamma and IL-10 in stimulated blood from dogs with clinical leishmaniosis. Parasites Vectors.

[bib31] Travi B.L., Cordeiro-da-Silva A., Dantas-Torres F., Miro G. (2018). Canine visceral leishmaniasis: diagnosis and management of the reservoir living among us. PLoS Negl. Trop. Dis..

[bib32] Ubeda J.M., Raymond F., Mukherjee A., Plourde M., Gingras H., Roy G. (2014). Genome-wide stochastic adaptive DNA amplification at direct and inverted DNA repeats in the parasite *Leishmania*. PLoS Biol..

[bib33] Van Bockstal L., Hendrickx S., Maes L., Caljon G. (2020). Sand fly studies predict transmission potential of drug-resistant *Leishmania*. Trends Parasitol..

[bib34] Van den Kerkhof M., Mabille D., Hendrickx S., Leprohon P., Mowbray C.E., Braillard S. (2020). Antileishmanial aminopyrazoles: studies into mechanisms and stability of experimental drug resistance. Antimicrob. Agents Chemother..

[bib35] Wagner V., Ibarra-Meneses A.V., Freitas T.R., Moreno J., Bhattacharya A., do Monte-Neto R.L., Fernandez-Prada C. (2025). Survival of the fittest: how *Leishmania* evades drug therapy. Adv. Parasitol..

[bib36] Yasur-Landau D., Jaffe C.L., David L., Baneth G. (2016). Allopurinol resistance in *Leishmania infantum* from dogs with disease relapse. PLoS Negl. Trop. Dis..

[bib37] Yasur-Landau D., Jaffe C.L., David L., Doron-Faigenboim A., Baneth G. (2018). Resistance of *Leishmania infantum* to allopurinol is associated with chromosome and gene copy number variations including decrease in the *S*-adenosylmethionine synthetase (*METK*) gene copy number. Int. J. Parasitol. Drugs Drug Resist..

